# Evolution of DNA ligases of Nucleo-Cytoplasmic Large DNA viruses of eukaryotes: a case of hidden complexity

**DOI:** 10.1186/1745-6150-4-51

**Published:** 2009-12-18

**Authors:** Natalya Yutin, Eugene V Koonin

**Affiliations:** 1 National Center for Biotechnology Information, National Library of Medicine, National Institutes of Health, Bethesda, MD 20894, USA

## Abstract

**Background:**

Eukaryotic Nucleo-Cytoplasmic Large DNA Viruses (NCLDV) encode most if not all of the enzymes involved in their DNA replication. It has been inferred that genes for these enzymes were already present in the last common ancestor of the NCLDV. However, the details of the evolution of these genes that bear on the complexity of the putative ancestral NCLDV and on the evolutionary relationships between viruses and their hosts are not well understood.

**Results:**

Phylogenetic analysis of the ATP-dependent and NAD-dependent DNA ligases encoded by the NCLDV reveals an unexpectedly complex evolutionary history. The NAD-dependent ligases are encoded only by a minority of NCLDV (including mimiviruses, some iridoviruses and entomopoxviruses) but phylogenetic analysis clearly indicated that all viral NAD-dependent ligases are monophyletic. Combined with the topology of the NCLDV tree derived by consensus of trees for universally conserved genes suggests that this enzyme was represented in the ancestral NCLDV. Phylogenetic analysis of ATP-dependent ligases that are encoded by chordopoxviruses, most of the phycodnaviruses and Marseillevirus failed to demonstrate monophyly and instead revealed an unexpectedly complex evolutionary trajectory. The ligases of the majority of phycodnaviruses and Marseillevirus seem to have evolved from bacteriophage or bacterial homologs; the ligase of one phycodnavirus, *Emiliana huxlei *virus, belongs to the eukaryotic DNA ligase I branch; and ligases of chordopoxviruses unequivocally cluster with eukaryotic DNA ligase III.

**Conclusions:**

Examination of phyletic patterns and phylogenetic analysis of DNA ligases of the NCLDV suggest that the common ancestor of the extant NCLDV encoded an NAD-dependent ligase that most likely was acquired from a bacteriophage at the early stages of evolution of eukaryotes. By contrast, ATP-dependent ligases from different prokaryotic and eukaryotic sources displaced the ancestral NAD-dependent ligase at different stages of subsequent evolution. These findings emphasize complex routes of viral evolution that become apparent through detailed phylogenomic analysis but not necessarily in reconstructions based on phyletic patterns of genes.

**Reviewers:**

This article was reviewed by: Patrick Forterre, George V. Shpakovski, and Igor B. Zhulin.

## Background

Viruses are ubiquitous parasites of all cellular life forms. In recent years, extensive genome sequencing and comparative analysis of both viral and host genomes yielded unprecedented insights into the evolution of viruses. In particular, it has been shown that 4 diverse families of large DNA viruses of eukaryotes (NCLDV), namely, Poxviridae, Asfarviridae, Iridoviridae, and Phycodnaviridae, share a set of conserved genes with functions implicated in replication, transcription and virion morphogenesis, suggesting an origin from a single ancestral virus. This apparently monophyletic class of viruses was denoted Nucleo-Cytoplasmic Large DNA Viruses (NCLDV) to emphasize the presence of a cytoplasmic stage in the reproduction of most if not all of these viruses [1]. The existence of such a cytoplasmic stage, during which virus replication is physically separated from the replication and expression of the host genome, and occurs in cytoplasmic viral "factories"[2,3], can be reasonably thought of as the driving force behind the retention of genes encoding replicative proteins in the NCLDV genomes. The analysis of subsequently sequenced genomes representing 3 additional viral families, namely, Ascoviridae, Mimiviridae, and very recently, a novel family typified by the Marseillevirus, strongly supported the conclusion on the monophyly of the NCLDV [4,5]. The reconstruction of the ancestral NCLDV gene set using a maximum parsimony method[4] or a more sophisticated maximum likelihood approach [6] led to the delineation of a set of 40-50 ancestral genes that include the genes for the key proteins required for genome replication, expression and virion morphogenesis.

One of the most dramatic revelations of comparative genomics is that the set of universal genes (defined in terms of orthologous gene sets) in all life forms is much smaller than the set of universal molecular functions [7,8]. The principal underlying cause is non-orthologous gene displacement (NOGD) whereby the same essential function is performed by unrelated or at least not orthologous genes [9]. Often, especially among prokaryotes, NOGD is coupled to horizontal gene transfer (HGT) of the respective genes. Owing to NOGD and HGT, phyletic patterns (that is, patterns of presence-absence in sequenced genomes) are complex, diverse and patchy for the majority of genes, even those involved in essential functions [10,11].

The case of viral genes is especially complicated because many viral functions can be complemented and replaced by functionally analogous host proteins that may or may not be homologous to the respective viral proteins; additionally, many viruses integrate host genes into their own genomes, so the functional repertoire of viral genes evolves in an incessant, dynamic interaction with the host gene repertoire. For instance, the RNA polymerase holoenzyme is obviously essential for the expression of any DNA genome. However, many large DNA viruses including certain NCLDV (such as some of the phycodnaviruses) that replicate in the cell nucleus (as well as herpesviruses and baculoviruses) do not encode any RNA polymerase subunits and fully rely on the host transcription machinery [4]. The DNA replication apparatus of the NCLDV is substantially autonomous from the host replication system. All sequenced NCLDV genomes contain genes for 3 essential replication enzymes, namely, DNA polymerase, primase and distinct replicative helicase (the latter two genes typically are fused to produce a two-domain primase-helicase). However, other proteins that are also essential for replication, in particular, DNA ligase and (predicted) flap endonuclease, are found only in subsets of the NCLDV, with the implication that viruses that lack these enzymes employ host analogs [4]. Very recently, this proposition was experimentally validated for the vaccinia virus (VACV) DNA ligase [12]. The DNA ligase gene of VACV can be knocked out with minimal or no adverse effect on virus growth in cell culture [13]. However, inhibition of the mammalian DNA ligase I but not any of the other 2 human ligases (III and IV) with a siRNA specifically abrogated the growth of the ligase knockout virus indicating that this host ligase was specifically recruited for VACV replication [12].

The DNA ligases of the NCLDV seem to epitomize the haphazard aspect of viral genome evolution. Some of the NCLDV encode ATP-dependent ligases that, among cellular life forms, are ubiquitous and essential for replication in archaea and eukaryotes, but are only sporadically found in bacteria where they seem to contribute to distinct forms of DNA repair [14-16]. Furthermore, the ligases of chordopoxviruses show extensive sequence similarity to mammalian ligase III [17]. Another subset of the NCLDV encode NAD-dependent ligases that are distantly related to the ATP-dependent ligases [18,19] and are ubiquitous and essential for replication in bacteria but represented only sporadically in archaea and eukaryotes [16,20]. Finally, a considerable fraction of the NCLDV do not appear to encode any ligases.

The reconstruction of the NCLDV genome evolution tentatively placed the ATP-dependent ligase into the last common ancestor, with the implication that in some lineages the putative ancestral ligase was lost, whereas in others it was replaced with the NAD-dependent ligases [4]. However, we were interested in applying phylogenetic methods along with comparative-genomic analysis, with the aim to reconstruct the history of the NCLDV ligases in greater detail and more definitively. The results of this study suggest an unexpected, complex evolutionary scenario.

## Results and Discussion

### Distribution of DNA ligases across the NCLDV genomes

We start with a census of DNA ligases encoded in the 45 currently available NCLDV genomes. Considering that some members of the ATP-dependent ligase family, particularly, in bacteria, show extreme sequence divergence [21], we searched all protein sequences of NCLDV with position-specific scoring matrices derived from multiple alignments of both ATP-dependent and NAD-dependent ligases. The ATP-dependent and NAD-dependent ligases show a perfect complementary pattern among the NCLDV, that is, not a single viral genome encodes both forms. Quantitatively, the ATP-dependent ligase is more common, being encoded by the majority of chordopoxviruses and phycodnaviruses, and the single available genomes of asfarvirus and Marseillevirus (Table 1). The distribution of the NAD-dependent ligases appears to be more scattered as they are encoded by entomopoxviruses and a single chordopoxvirus (Crocodilepox virus that, however, encodes a truncated version of the NAD-dependent ligase that may or may not be active), mimi/mamaviruses, and a minority of iridoviruses (Table 1). Viruses lacking any ligase gene are found in all extensively sampled NCLDV families, namely, poxviridae, iridoviridae, ascoviridae (so far none of the sequenced genomes in this family has a gene for a ligase), and phycodnaviridae (Table 1), suggesting multiple losses during evolution of the NCLDV.

**Table 1:** DNA ligases of the NCLDV

Viral family	Subfamily/genus	Species	ATP-dependent ligase (GI/gene name)	NAD-dependent ligase (GI/gene name)
**Poxviridae**	**Chordopoxvirinae**			

	Avipoxvirus	Canarypox virus	40555999	

		Fowlpox virus	9634713/FPV043	

	Capripoxvirus	Goatpox virus Pellor	148913010	

		Sheeppox virus 17077-99	21492584	

		Lumpy skin disease virus NI-2490	15150572	

	Cervidpoxvirus	Deerpox virus W-848-83	62637522	

	Leporipoxvirus	Myxoma virus	9633769	

		Rabbit fibroma virus	9633943	

	Molluscipoxvirus	Molluscum contagiosum virus		

	Orthopoxvirus	Vaccinia virus	66275973/A50R	

		Variola virus (smallpox virus)	9627683/J4R	

	Parapoxvirus	Orf virus, complete genome		

		Bovine papular stomatitis virus		

	Suipoxvirus	Swinepox virus	18640216	

	Yatapoxvirus	Tanapox virus		

		Yaba monkey tumor virus		

		Yaba-like disease virus		

	unclassified Chordopoxvirinae	Crocodilepox virus		115531716^a^

	**Entomopoxvirinae**	Amsacta moorei entomopoxvirus		9964513/AMV199

		Melanoplus sanguinipes entomopoxvirus		9631366/MSV162

**Ascoviridae**	**Ascovirus**	Heliothis virescens ascovirus 3e		

		Trichoplusia ni ascovirus 2c		

		Spodoptera frugiperda ascovirus 1a		

**Asfarviridae**	**Asfavirus**	African swine fever virus	9628207/NP419L	

**Iridoviridae**	**Chloriridovirus**	Aedes taeniorhynchus iridescent virus (Invertebrate iridescent virus 3)		109287930

	**Iridovirus **(small iridescent insect viruses)	Invertebrate iridescent virus 6		15078917/CIV205R

	**Lymphocystivirus**	Lymphocystis disease virus 1		

		Lymphocystis disease virus - isolate China		

	**Megalocytivirus**	Infectious spleen and kidney necrosis virus		

	**Ranavirus**	Singapore grouper iridovirus		

		Frog virus 3		

		Ambystoma tigrinum virus		

**Mimiviridae**	**Mimivirus**	Acanthamoeba polyphaga mimivirus		55819181/MIMI_R303

	**Mamavirus**	Mamavirus		unpublished

**Phycodna-viridae**	**Chlorovirus**	Paramecium bursaria Chlorella virus AR158	157953848	

		Paramecium bursaria Chlorella virus NY2A	157953038	

		Paramecium bursaria chlorella virus MT325		

		Acanthocystis turfacea Chlorella virus 1	155371134	

		Paramecium bursaria Chlorella virus FR483		

		Paramecium bursaria Chlorella virus 1	9632109/A544R	

	**Coccolithovirus**	Emiliania huxleyi virus 86	73852627/EhV158	

	**Phaeovirus**	Feldmannia species virus		

		Ectocarpus siliculosus virus 1		

	**unclassified Phycodnaviridae**	Ostreococcus virus OsV5	163955177	

**Marseille virus**		Marseille virus	Unpublished	

^a^A truncated protein missing OB-fold and HhH (helix-hairpin-helix) domains.

### Phylogenies of the DNA ligases of the NCLDV

**Figure 1:**
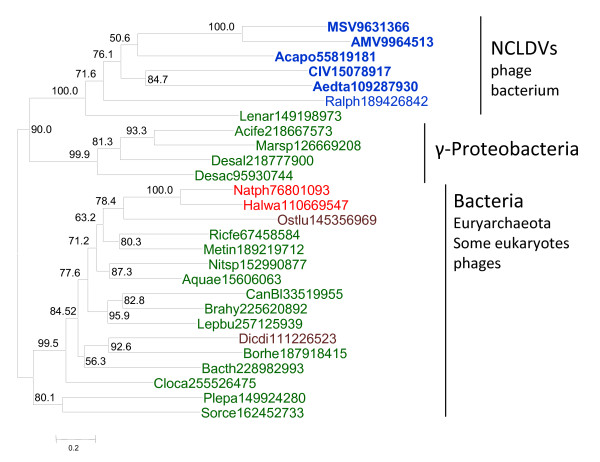
**A maximum-likelihood phylogenetic tree of NAD-dependent DNA ligases**. The numbers indicate the statistical support (Expected-Likelihood Weights) of internal nodes. The support values are given as percentages (n = 1,000). Archaeal sequences are color-coded red, bacterial sequences green, eukaryotic sequences brown, and viral sequences blue. The NCLDV are shown in bold type. All proteins are denoted by their Genbank identification numbers (GIs). The truncated sequence of the NAD-dependent ligase of Crocodilepox virus was not used for phylogenetic analysis. Abbreviations: MSV, *Melanoplus sanguinipes *entomopoxvirus; AMV, *Amsacta moorei *entomopoxvirus; Acapo, *Acanthamoeba polyphaga *mimivirus; CIV, Chilo iridescent virus (Invertebrate iridescent virus 6); Aedta, *Aedes taeniorhynchus *iridescent virus (Invertebrate iridescent virus 3); Ralph, *Ralstonia solanacearum *phage RSL1; Lenar, *Lentisphaera araneosa *HTCC2155; Acife, *Acidithiobacillus ferrooxidans *ATCC 23270; Marsp, *Marinobacter *sp. ELB17; Desal, *Desulfatibacillum alkenivorans *AK-01; Desac, *Desulfuromonas acetoxidans *DSM 684; Natph, *Natronomonas pharaonis *DSM 2160; Halwa, *Haloquadratum walsbyi *DSM 16790; Ostlu, *Ostreococcus lucimarinus *CCE9901; Ricfe, *Rickettsia felis *URRWXCal2; Metin, *Methylacidiphilum infernorum *V4; Nitsp, *Nitratiruptor *sp. SB155-2; Aquae, *Aquifex aeolicus *VF5; CanBl, *Candidatus Blochmannia floridanus*; Brahy, *Brachyspira hyodysenteriae *WA1; Lepbu, *Leptotrichia buccalis *DSM 1135; Dicdi, *Dictyostelium discoideum *AX4; Borhe, *Borrelia hermsii *DAH; Bacth, *Bacillus thuringiensis *Bt407; Cloca, *Clostridium carboxidivorans *P7; Plepa, *Plesiocystis pacifica *SIR-1; Sorce, *Sorangium cellulosum*.

To reconstruct the evolutionary scenario for viral ligases, we used multiple alignments of the NAD-dependent and ATP-dependent ligases that included the respective protein sequences from the NCLDV, other viruses, and representative archaea, bacteria, and eukaryotes (see Additional File 1 and Additional File 2, respectively), to build maximum likelihood (ML) phylogenetic trees. The tree for the NAD-dependent ligases contains an unequivocally supported NCLDV clade (Figure 1). Statistical evaluation of alternative tree topologies using the Approximately Unbiased (AU) test [22] indicated that trees with polyphyletic NCLDV effectively could be ruled out (all these alternative topologies had zero AU values). The NCLDV clade belonged to a branch that included mostly NAD-dependent ligases from gamma-proteobacteria along with some bacteriophage ligases one of which clustered with the NCLDV (Figure 1).

**Figure 2:**
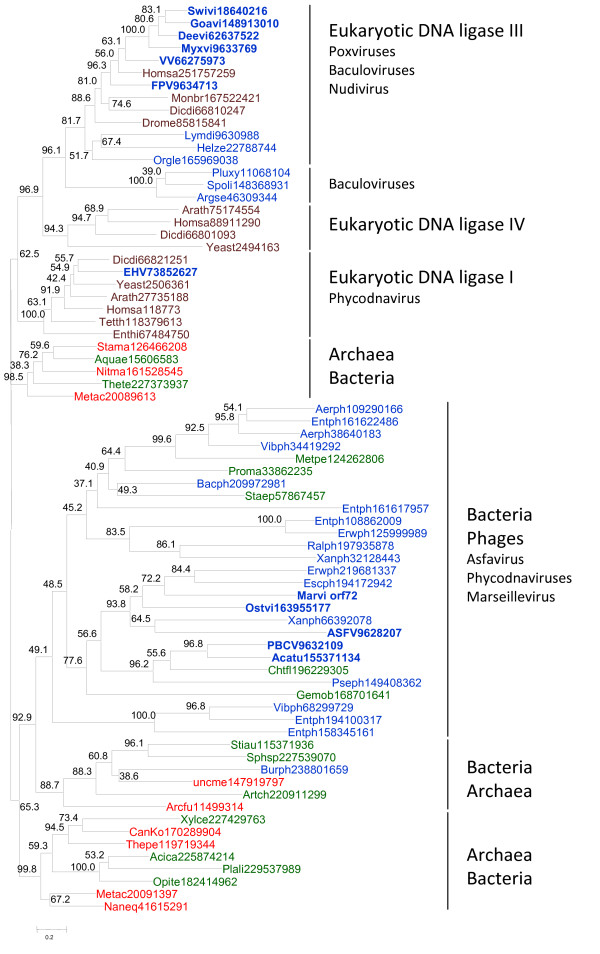
**A maximum-likelihood phylogenetic tree of ATP-dependent DNA ligases**. The designations are as in Figure 1. For a tree with an extended set of vertebrate sequences, see Additional File 4. Abbreviations: Acatu, *Acanthocystis turfacea Chlorella *virus 1; Acica, *Acidobacterium capsulatum *ATCC 51196; Aerph, *Aeromonas *phage; ASFV, African swine fever virus; Aquae, *Aquifex aeolicus *VF5; Arath, *Arabidopsis thaliana*; Arcfu, *Archaeoglobus fulgidus *DSM 4304; Argse, *Agrotis segetum *granulovirus; Artch, *Arthrobacter chlorophenolicus *A6; Bacph, *Bacillus *phage SPO1; Burph, *Burkholderia *phage BcepIL02; CanKo, *Candidatus Korarchaeum cryptofilum *OPF8; Chtfl, *Chthoniobacter flavus *Ellin428; Deevi, *Deerpox *virus W-848-83; Dicdi, *Dictyostelium discoideum *AX4; Drome, *Drosophila melanogaster*; EHV, *Emiliania huxleyi *virus 86; Enthi, *Entamoeba histolytica *HM-1:IMSS; Entph, *Enterobacteria *phage; Erwph, *Erwinia *phage; Escph, *Escherichia *phage rv5; FPV, *Fowlpox *virus; Gemob, *Gemmata obscuriglobus *UQM 2246; Goavi, *Goatpox *virus Pellor; Helze, *Heliothis zea *virus 1; Homsa, *Homo sapiens*; Lymdi, *Lymantria dispar *MNPV; Marvi, Marseillevirus; Metac, *Methanosarcina acetivorans *C2A; Metpe, *Methylibium petroleiphilum *PM1; Monbr, *Monosiga brevicollis *MX1; Myxvi, *Myxoma *virus; Naneq, *Nanoarchaeum equitans *Kin4-M; Nitma, *Nitrosopumilus maritimus *SCM1; Opite, *Opitutus terrae *PB90-1; Orgle, *Orgyia leucostigma *NPV; Ostvi, *Ostreococcus *virus OsV5; PBCV, *Paramecium bursaria Chlorella *virus 1; Plali, *Planctomyces limnophilus *DSM 3776; Pluxy, *Plutella xylostella *granulovirus; Proma, *Prochlorococcus marinus*; Pseph, *Pseudomonas *phage F8; Ralph, *Ralstonia *phage RSB1; Sphsp, *Sphingobacterium spiritivorum *ATCC 33300; Spoli, *Spodoptera litura *granulovirus; Staep, *Staphylococcus epidermidis *RP62A; Stama, *Staphylothermus marinus *F1; Stiau, *Stigmatella aurantiaca *DW4/3-1; Swivi, *Swinepox *virus; Tetth, *Tetrahymena thermophila*; Thepe, *Thermofilum pendens *Hrk 5; Thete, *Thermobaculum terrenum *ATCC BAA-798; VV, *Vaccinia *virus; Vibph, *Vibrio *phage; Xanph, *Xanthomonas *phage; Xylce, *Xylanimonas cellulosilytica *DSM 15894; Yeast, *Saccharomyces cerevisiae*.

In a sharp contrast, the tree of ATP-dependent ligases showed a scattered distribution of the NCLDV branches (Figure 2). The chordopoxvirus ligases formed a strongly supported clade with eukaryotic ligase III as suggested by the previously noticed high sequence similarity between these proteins [17]. Although it has been claimed that ligase III was unique to vertebrates [14], our analysis detected clear orthologs in insects, the choanoflaggelate *Monosiga brevicola *and the social amoeba *Dictyostelium discoideum*, suggesting that ligase III evolved through a duplication of the ligase IV gene at the onset of evolution of unikonts but was repeatedly lost in fungi and several animal lineages (Figure 2 and Additional File 3). The NCLDV ligases clustered with the vertebrate homologs to the exclusion of the homologs from other unikonts (Figure 2 and Additional File 4). Interestingly, several non-NCLDV animal viruses with large DNA genomes from the families Baculoviridae and Nudiviridae also encode a ligase related to ligase III (Figure 2).

The ATP-dependent ligase of one of the phycodnaviruses, *Emiliana huxlei *virus (representative of the genus *Coccolithovirus*), belonged to the eukaryotic ligase I branch (Figure 2). The rest of the ATP-dependent ligases of the NCLDV, namely, those of African Swine Fever virus (the only available representative of the family Asfarviridae), phycodnaviruses and Marseillevirus, were scattered within a large cluster of ATP-dependent ligases of various bacteria and bacteriophages (Figure 2). The AU test indicated that the tree in which the latter 3 groups of viral ligases formed a clade could be effectively ruled out as an likely alternative to the tree in Figure 2 (the alternative topology with monophyletic NCLDV was associated with a zero AU value).

### Evolutionary scenario for the DNA ligases of the NCLDV

**Figure 3:**
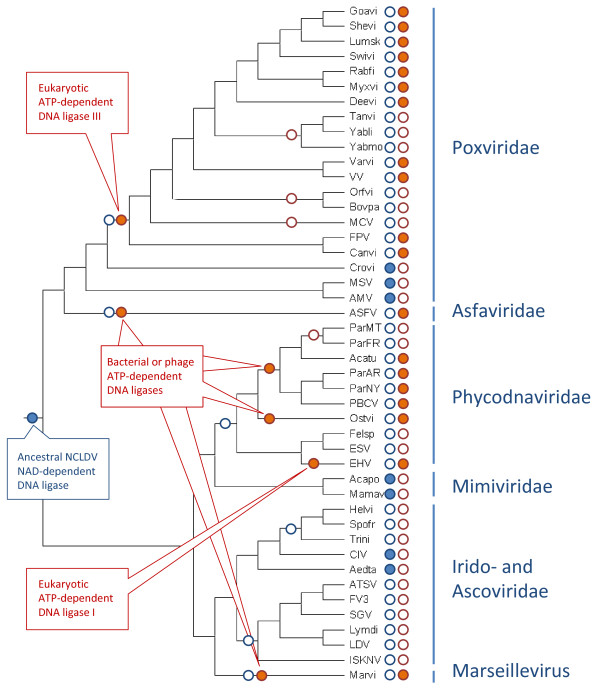
**Evolutionary scenario for the DNA ligases of the NCLDV**. The underlying species tree is the consensus of maximum-likelihood phylogenetic trees of 4 universal NCLDV genes (see text)[6]. Filled circles denote presence (in extant viruses) or acquisition (in ancestral forms) and empty circles denote absence or loss of the respective ligases (blue: NAD-dependent ligase, red: ATP-dependent ligase). Abbreviations: Canvi, Canarypox virus; FPV, Fowlpox virus; Goavi, Goatpox virus Pellor; Shevi, Sheeppox virus 17077-99; Lumsk, Lumpy skin disease virus NI-2490; Deevi, Deerpox virus W-848-83; Myxvi, Myxoma virus; Rabfi, Rabbit fibroma virus; MCV, *Molluscum contagiosum *virus; VV, Vaccinia virus; Varvi, Variola virus (smallpox virus); Orfvi, Orf virus; Bovpa, Bovine papular stomatitis virus; Swivi, Swinepox virus; Tanvi, Tanapox virus; Yabmo, Yaba monkey tumor virus; Yabdi, Yaba-like disease virus; Crovi, Crocodilepox virus; AMV, *Amsacta moorei *entomopoxvirus; MSV, *Melanoplus sanguinipes *entomopoxvirus; Helvi, *Heliothis virescens *ascovirus 3e; Trini, *Trichoplusia ni *ascovirus 2c; Spofr, *Spodoptera frugiperda *ascovirus 1a; ASFV, African swine fever virus; Aedta, *Aedes taeniorhynchus *iridescent virus (Invertebrate iridescent virus 3); CIV, Invertebrate iridescent virus 6; Lymdi, Lymphocystis disease virus 1; LDV, Lymphocystis disease virus - isolate China; ISKNV, Infectious spleen and kidney necrosis virus; SGV, Singapore grouper iridovirus; FV3, Frog virus 3; ATSV, *Ambystoma tigrinum *virus; Acapo, *Acanthamoeba polyphaga *mimivirus; Mamav, Mamavirus; ParAR, *Paramecium bursaria *Chlorella virus AR158; ParNY, *Paramecium bursaria *Chlorella virus NY2A; ParMT, *Paramecium bursaria *chlorella virus MT325; Acatu, *Acanthocystis turfacea *Chlorella virus 1; ParFR, *Paramecium bursaria *Chlorella virus FR483; PBCV, *Paramecium bursaria *Chlorella virus 1; EHV, *Emiliania huxleyi *virus 86; Felsp, Feldmannia species virus; ESV, *Ectocarpus siliculosus *virus 1; Ostvi, Ostreococcus virus OsV5; Marvi, Marseille virus.

To reconstruct the evolutionary history of the NCLDV ligases, we combined 3 lines of evidence: i) phyletic distribution of the ATP-dependent and NAD-dependent ligases among the NCLDV, ii) topologies of the phylogenetic trees for the NAD-dependent ligases (Figure 1) and ATP-dependent ligases (Figure 2), iii) the "species tree" of the NCLDV for which we used the consensus of the trees for conserved NCLDV genes (Figure 3) [6]. Of these, the species tree is arguably the weakest link given that there are few genes that are conserved in all NCLDV, and the topologies of the individual trees of these genes are not identical, so a consensus had to be derived to produce the tree topology in Figure 3[6]. Nevertheless, the topology of the consensus tree is mostly compatible with that of a tree derived by comparison of phyletic patterns, suggesting that the consensus tree is a reasonable representation of a central trend in the evolution of the NCLDV genomes [6]. Under this assumption, we superimposed the phyletic patterns of the ATP-dependent and NAD-dependent ligases onto the species tree and invoked the tree topologies of both ligases to infer the evolutionary scenario (Figure 3). Under this scenario, the ancestral NCLDV possessed a gene for an NAD-dependent ligase that was replaced with an ATP-dependent ligase on multiple, independent occasions. Formally, an alternative hypothesis [23] cannot be ruled out, namely, that the NAD-dependent ligase gene was originally acquired by one of the NCLDV lineages, perhaps, the mimiviruses which replicate in amoebae where gene exchanges between viral and bacterial parasites and symbionts appear to be common [5,24,25]. This scenario implies that the ancestral NCLDV encoded no ligase, like many extant viruses. However, the monophyly of the NAD-dependent ligases from 3 distinct NCLDV lineages (Figure 1), taken together with the polyphyly of the ATP-dependent ligases (Figure 2), favors the scenario of ancestral capture of the NAD-dependent ligase (Figure 3). Indeed, the alternative would require multiple gene transfers between viruses infecting phylogenetically distant hosts, apparently, not a particularly plausible possibility.

The specific sources of the NCLDV ligases are difficult to pinpoint with certainty but the trees in Figures 1 and 2 provide clues. The clustering of NAD-dependent viral ligases with homologs from (primarily) gamma-proteobacteria and bacteriophages (Figure 1) suggests that the viral ancestor of the NCLDV captured the ligase gene from a bacteriophage, perhaps, one that infected the mitochondrial endosymbiont. This scenario is compatible with the hypothesis that the NCLDV evolved concomitantly with eukaryogenesis [4]. Indeed, the acquisition of the ligase gene was most likely an early event considering the rapid degradation of the mitochondrial endosymbiont [26]. The tree of ATP-dependent ligases (Figure 1) suggests that they displaced the ancestral NAD-dependent ligase on several independent occasions and at different stages of viral evolution (Figure 3). Early replacements of the ancestral NAD-dependent ligase by ATP-dependent ligases of bacterial or bacteriophage origin apparently occurred independently in phycodnaviruses, Marseillevirus, and asfarviruses. By contrast, later displacements of the NAD-dependent ligases with ATP-dependent ligases of eukaryotic origin seem to have occurred in *Emiliana huxlei *virus and in chordopoxviruses. In particular, ligase III apparently was acquired by chordopoxviruses not only after the radiation of the entompoxviruses and chordopoxviruses (probably concomitant with the radiation of the host animals) but some time into the evolution of chordopoxviruses themselves, given that the earliest branching group in this subfamily, crocodile pox virus, still has an NAD-dependent ligase (Table 1 and Additional File 1). Considering the absence of genes for any ligases in many extant NCLDV and the lack of genomes encoding both an NAD-dependent and an ATP-dependent ligase, it seems likely that the displacements occurred via a ligase-less intermediate as opposed to an intermediate encoding both types of ligases.

Both the major form of bacterial and bacteriophage NAD-dependent ligase (LigA) and eukaryotic ligase III possess a C-terminal BRCT (BRCA1 C-terminal) domain that mediates interactions with components of various protein complexes involved in repair and cell cycle control[27,28]. The BRCT domain is present in the NAD-dependent ligases of the mimiviruses and iridoviruses but is missing in the NAD-dependent ligases of entomopoxviruses and the ATP-dependent ligases of chordopoxviruses, the apparent origin of the latter from ligase III notwithstanding. Thus, acquisition of cellular ligase genes by distinct NCLDV lineages was accompanied by independent but analogous truncations of the acquired gene.

## Concluding remarks

The phylogenomic analysis described here led to unexpected conclusions. Although ATP-dependent ligases are more common among the NCLDV than NAD-dependent ligases, it is the latter that can be traced back to the last common ancestor of the NCLDV whereas the ATP-dependent ligases apparently were acquired by several viral lineages at different stages of their evolution. The most general message brought about by these findings is that phyletic patterns alone, at least, in some cases, are insufficient for an accurate evolutionary reconstruction and can lead to substantially oversimplified or even false scenarios.

More specifically, the study of the evolution of viral ligases has general implications for understanding the evolution of the NCLDV. The apparent acquisition of the NAD-dependent ligase gene at an early stage of evolution antedating the last common ancestor of the NCLDV is compatible with the scenario of the origin of eukaryotic viruses by assembly of genes from diverse sources including bacteriophages and bacteria in the course of eukaryogenesis [29]. The apparent independent displacement of the NAD-dependent ligase by ATP-dependent ligases of bacterial/bacteriophage origin in several NCLDV lineages implies that the primary radiation of the NCLDV occurred at the earliest stages of the evolution of eukaryotes, conceivably, prior to the radiation of eukaryotic supergroups and before the process of mitochondrial reduction was completed. A similar interpretation of the NCLDV evolution was given previously on the basis of the examination of the host ranges of the major lineages of these viruses [4]. Phylogenomic analysis of other conserved NCLDV genes has the potential to further clarify the evolutionary scenario and, possibly, the origin of this important class of viruses.

## Methods

The non-redundant protein sequence databases at the NCBI were searched using the BLASTP and PSI-BLAST programs with the expectation (E) value for sequence inclusion in PSI-BLASt iterations set at 0.005 [30]. The sequences for phylogenetic analysis were aligned using the MUSCLE program with the default parameters [31]. To eliminate poorly aligned regions, each alignment column was assigned a homogeneity value by scaling the sum-of-pairs score within the column between those of a homogeneous column (the same residue in all aligned sequences) and a random column (YIW, I. A. Seledtsov, K. S. Makarova, unpublished). Columns with homogeneity of less than 0.2 and/or with more than one-third of gap characters were removed from the alignment.

Maximum Likelihood (ML) phylogenetic trees were constructed using the TreeFinder software [32], with the estimated site rates heterogeneity and the WAG (Whelan and Goldman) substitution model [33]. The Expected-Likelihood Weights [34] of 1,000 local rearrangements were used as confidence values of TreeFinder tree branches. The Approximately Unbiased (AU) test of tree topologies [22] was applied using TreeFinder [32].

## Competing interests

The authors declare that they have no competing interests.

## Authors' contributions

EVK initiated and planned the study, and wrote the manuscript; NY analyzed data; the final manuscript was read and approved by both authors.

## Reviewers' reports

### Reviewer 1: Patrick Forterre, Institut Pasteur

The origin of viral genes is presently a hot topic of discussion. Some authors consider that all viral genes originated from cellular genes robbed by viruses whereas others emphasize that new genes can also appear in viral lineages and be transferred later on from viruses to cells. Phylogenies combining viral and cellular genes are indeed often difficult to interpret, especially the direction of transfer, and the interpretation can be strongly dependent of the prejudice of the author. This paper is an example of such case. Eugene Koonin has recognized in a previous paper the existence of specific viral genes (hallmark genes) that, according to him, even originated before cells, in a primordial hydrothermal vent [29]. However, he is also a proponent of the idea that eukaryotes and their viruses originated after the emergence of Archaea and Bacteria from this primordial vent [29]. In this scenario, eukaryotes originated by the association of an ancient bacterium and an ancient archaeon, and eukaryotic viruses originated by combining genes of bacterial and archaeal viruses together with eukaryotic genes from their hosts. In this scenario, genes present in eukaryotic viruses could not have originated directly from the ancestral viral world, but should have originated either from prokaryotic viruses or from the virus hosts. As a consequence, the authors systematically favour in this paper transfers from cells to viruses to explain the origin of NCLDV ligases.

I have a different prejudice. I think that most core genes of NCLDV are viral hallmark genes that originated directly in ancient viral lineages. Furthermore, I suspect that NCLDV proteins involved in replication might have played an important role in the origin of modern eukaryotic DNA genomes [35]. I thus favour the possibility of ancient LGT from NCLDV to eukaryotes. The phylogenies presented in this paper do not really allow to decide between these different interpretations but raises interesting questions.

An important point, in my opinion, is to determine, if possible, which ligase (s) was (were) present in the last common ancestor of each of the three domains. This is missing in the manuscript. To my understanding, the ancestral bacterium should have contained an NAD-dependent DNA ligase. It should thus be important to present a more exhaustive tree of bacterial NAD ligase in Figure 1, with their distribution among the various bacterial divisions, to clarify this point. For me, the tree in Figure 1 suggests that several subfamilies of NAD ligases were already established before one of them was recruited in the bacterial domain. This tree suggests also the existence of several subfamilies presently encoded by bacterioviruses and distantly related to the NCLDV DNA ligases. For me, it fits very well with the idea that the bacterial DNA ligase has a viral origin, and that Bacteria on one side, Archaea/Eukarya on the other, recruited independently their DNA replication machinery from different viruses.

In the case of ATP-dependent ligases, it should be important to determine which ligases were present in the LECA (the Last Eukaryotic Common Ancestor), in the ancestor of Archaea, and possibly in the common ancestor of Archaea and Eukarya. The trees suggest for me that ATP-dependent ligases were in fact recruited several times independently in the three domains. It should be also important to indicate on the tree where are the hosts of respective viruses. This can sometimes helps to polarize the direction of transfer. For instance, if the host is not located at its expected position in the cellular tree, one can suspect a transfer from the virus to the host.

In these phylogenies, when the authors detect a DNA ligase gene in Bacteria or Archaea, it would be important to determine if this is the *bona fide *ligase of the domain, or a ligase present in the genome of an integrated virus and/or plasmid [36]. In other words, it is important to distinguish in cellular genomes those genes that are really cellular (they were already present in the ancestor of a particular domain or recruited from another cell by LGT) and those that are in fact viral (present in integrated viruses or related elements). The confusion between these two kinds of genes in phylogenies can explain, in my opinion, the present confusion between networks and trees.

Finally, it should be interesting to have an idea of the structural differences between the various ligase subfamilies studied here. In my opinion, cellular ligases (originating from the ancestor of a particular domain) should be very similar in terms of structure, as it is the case for instance for DNA gyrase in all Bacteria or Topo II in all Eukarya, or else for Topo IB in both Archaea and Eukarya [37,38]. In contrast, families that diverged from ancient viral lineages before LUCA should exhibit more structural differences (as it is the case between DNA gyrase, T4 and eukaryotic Topo II or else between archaeal/eukayal Topo IB and bacterial or Poxvirus Topo IB) [37,38].

I thus encourage the authors to consider the two alternative possibilities in the discussion of their results, either LGT from cells to viruses or from viruses to cells.

#### Authors' response

*We greatly appreciate this review that puts the rather limited and specific study described in this article into the much more general context of evolution of viruses and cells, and while doing so, offers a perspective on these general issues that is orthogonal to our own view in some important aspects while congruent in others. There is no need to discuss these concepts as this was done in several previous publications *[29,39,40]. *However, a brief summary of the differences is due. The distinction between our position and that of Forterre that is of primary relevance for the conclusions of this study is the adoption of different scenarios for the origin of eukaryotes. Our position is that the first eukaryotic cells emerged as a result of engulfment of an alpha-proteobacterium, the future mitochondrion, by an archaeon. To the best of our understanding, this scenario is best compatible both with comparative-genomic results and with more general considerations stemming from the parsimony principle *[41-44]. *This scenario, of course, has critical implications for the origin of viruses infecting eukaryotes: these viruses are thought to have evolved in a « second melting pot of viral evolution », concomitant with eukaryogenesis, through amalgamation of gene from viruses of prokaryotes, bacteria, archaea and the emerging eukaryote *[29]. *Under this scenario, the possibility of the origin of any genes of the NCLDV directly from the primoridal pool of virus-like elements can be safely ruled out although some of the hallmarks could come from that pool through viruses of prokaryotes, having never been integral genomic components of cellular life forms. Acquisition of genes from the evolving NCLDV by the eukaryotic host remains a possibility but there seems to be no compelling evidence that this was a major route of eukaryote evolution*.

*Forterre propounds a different scenario of evolution that includes a primordial virus world as well but considers eukaryotes to be one of the primary lines of descent in the evolution of cells *[35,45-47]. *It is further proposed that original bacterial, archaeal and eukaryotic cells might have possessed RNA genomes, whereas the DNA replication machineries were invented by viruses and independently grafted onto the 3 cell types *[35]. *Other authors also have developed evolutionary scenarios under which eukaryotes represent a primordial cellular lineage, possibly, even the first type of cells to evolve *[48,49]. *In our view, there is little evidence if any evidence in of the « primordial eukaryotes » scenarios (see specifically *[50]), *so we cannot really agree that our adherence to the symbiogenetic scenario is a « prejudice ». Nevertheless, we do recognize that a definitive elucidation of the sequence of events that led to the emergence of eukaryotes is an extremely difficult task that requires much additional phylogenomic analysis and might not be attainable in the near future. Clearly, under the « primordial eukaryotic lineage » scenario, viral hallmark genes in the NCLDV could plausibly originate from the primordial virus world, and transfer of genes from these viruses to the eukaryotic hosts potentially could be a major route of evolution. Thus, interpretation of the phylogenomic analysis of viral genes, to a large extent, hinges on the choice between the two orthogonal scenarios for the origin of eukaryotes that cannot be definitively distinguished at this time*.

*Having acknowledged this uncertainty, we would like to point out that the history of the NCLDV ligases elucidated in this study is poorly compatible with the contribution of viral genes to the host genomes that is hypothesized by Forterre. Indeed, we show that viral NAD-dependent ligases are monophyletic and by implication were probably present in the common ancestor of the extant NCLDV. However, this class of ligases is only sporadically represented in eukaryotic genomes (it cannot be ruled out that the respective genes were acquired from viruses although so far there are no direct evidence of such transfers). By contrast, ATP-dependent ligases are ubiquitous in eukaryotes but polyphyletic in the NCLDV, a finding that seems to effectively rule out the origin of eukaryotic ligases from viruses of this class. Moreover, there are two « smoking » guns of acquisition of ATP-dependent ligase genes from the hosts by distinct lineages of the NCLDV, namely, the ligase III homolog in poxviruses and the ligase I homolog in Emiliana huxlei *virus.

*Finally, we should note that a more extensive (comprehensive) phylogenetic analysis suggested by Forterre is certainly of interest. However, in this paper, we focus specifically on the history of the NCLDV ligases; for this analysis, we used representative sets of ATP-dependent and NAD-dependent ligases from bacteria (and bacterial viruses), archaea, and eukaryotes, so we do not expect that inclusion of more exhaustive sequence sets (which complicates the construction of ML trees) would affect the message*.

### Reviewer 2: George V. Shpakovski, Shemyakin-Ovchinnikov Institute of Bioorganic Chemistry, Russian Academy of Sciences, Moscow, Russia

The authors have performed a detailed phylogenomic analysis and reconstructed the history of the DNA ligases in Nuclear-Cytoplasmic Large DNA Viruses of eukaryotes (NCLDV). The study reveals a quite complex evolutionary trajectory of the enzyme involved in NCLDV' genome replication: although ATP-dependent ligases are approx. 3 times more often present in different NCLDVs and previous studies (Ref. 1 & Ref. 4 in the paper) regarded this type of enzyme as the one which was present in the ancestral NCLDV genome (alone or together with the NAD-dependent DNA ligase form), the last common ancestor of the NCLDV probably contained an NAD-dependent enzyme only (Fig. 3). The novel conclusion reported in the manuscript is based mostly on the monophyly of both the NCLDV as a group and all viral NAD-dependent ligases. The story is nicely presented and interpreted but, in my opinion, the scenario suggested is not the only one which could be inferred. Because of clearly demonstrated polyphyletic origin of the viral ATP-dependent ligases (Fig. 2), the presence of enzyme of this type in the ancestral NCLDV genome can be effectively ruled out. But the fourth remaining possibility (that the ancient, primordial NCLDV genome did not contained its own DNA ligase, but employed host analogs instead, like 19 out of 45 currently known viruses do) cannot be excluded. Following this assumption it could be possible to effectively decrease the total number of evolutionary events (exemplified by 'lost' or 'gain' of the DNA ligases - see Fig. 3 of the manuscript) from 17 events (as it is now in the authors' interpretation) to only 10, which will be more in concord with the Occam's razor principle. Additional data and further research will probably clarify the matter.

#### Authors' response

*The hypothesis that the ancestral NCLDV encoded no ligase was implicit in the original manuscript but in the revision we made it explicit. However, we also indicate that this scenario is not particularly plausible, given the monophyly of the NAD-dependent ligases of the NCLDV, because it would require multiple gene transfers between viruses infecting taxonomically distant hosts. Put another way, although the number of the events under this scenario indeed could be smaller than under the ancestral NAD-dependent ligase scenario that we favor, the nature of the inferred events also should be taken into account. As some classes of events (such as the well established gene loss) are more likely than other (such as the dubious gene transfer between viruses from distant hosts)*.

### Reviewer 3: Igor Zhulin, Joint Institute for Computational Sciences, University of Tennessee - Oak Ridge National Laboratory

This is a study of DNA ligases in Eukaryotic Nucleo-Cytoplasmic Large DNA Viruses (NCLDV). Unlike other viruses, which exclusively use the host DNA replication machinery, many of NCLDV encode their own DNA replication machinery. This machinery proves useful when viruses inhabit the cytoplasm of eukaryotes, rather than the nucleus. NCLDV have been shown to encode two types of DNA ligases: NAD-dependent and ATP-dependent. ATP-dependent ligases were known to be more prevalent, but the details of their origins were unknown. Bacteria predominantly use NAD-dependent ligases, whereas Archaea and Eukaryotes primarily use ATP-dependent ligases. Here authors collected the NAD and ATP ligases from NCLDV genomes and examined their distribution patterns as well as their relationships to homologous enzymes in selected Bacteria, Archaea, and Eukaryotes. Phylogenetic analysis of the proteins shows that all viral NAD-dependent ligases are monophyletic, whereas viral ATP-dependent enzymes show sporadic distribution throughout the trees. This information along with distribution patterns mapped onto a viral species tree built from conserved NCLDV genes support that NAD-dependent ligases were present in the NCLDV common ancestor, but were displaced by ATP-dependent ligases on multiple independent occasions in viral evolution.

This is a very clearly organized manuscript. It addresses a specific question, and the results support the conclusions. I'm not as excited about the results as are the authors, but that is because I do not know much about viruses.

I have one minor question that is more of a personal curiosity than a criticism. The species tree (Fig. 3) is a consensus of the individual trees of conserved NCLDV genes, and I wonder how this would compared to a tree built from a concatenated alignment of these genes/proteins?

#### Authors' response

This issue is addressed elsewhere [5,6]. The differences in the tree topologies are minor.

My only issue with the paper is the methods section on sequence identification and alignment. It is too short and contains no detail whatsoever. There is nothing I can say that is not illustrated by simply copying it below:

"Protein sequence databases at the NCBI were searched using the BLASTP and PSIBLAST programs [30]. The sequences for phylogenetic analysis were aligned using the MUSCLE program [31]. Poorly conserved positions and positions including gaps in more than one-third of the sequences were removed prior to tree computation".

What databases? What cutoffs and parameters? How were the poorly conserved positions identified?

Providing the necessary methodology details is essential, especially for a computational paper.

Authors' response: *The details are included in the revised manuscript*.

## Supplementary Material

Additional file 1 Multiple sequence alignment of NAD-dependent ligases used for phylogenetic analysis. Click here for file

Additional file 2 Multiple sequence alignment of ATP-dependent ligases used for phylogenetic analysis. Click here for file

Additional file 3 A maximum-likelihood phylogenetic tree of fungal ATP-dependent DNA ligases. Click here for file

Additional file 4 A maximum-likelihood phylogenetic tree of ATP-dependent DNA ligases including multiple vertebrate species. Click here for file
