# Supplementary material for: Evolution of DNA ligases of Nucleo-Cytoplasmic Large DNA viruses of eukaryotes: a case of hidden complexity
Source: Biol Direct. 2009 Dec 18;4:51. doi: 10.1186/1745-6150-4-51 (PMC2806865; doi:10.1186/1745-6150-4-51)
Supplement: Additional file 3 — A maximum-likelihood phylogenetic tree of fungal ATP-dependent DNA ligases. [file 1745-6150-4-51-S3.PPT]

## Slide 1
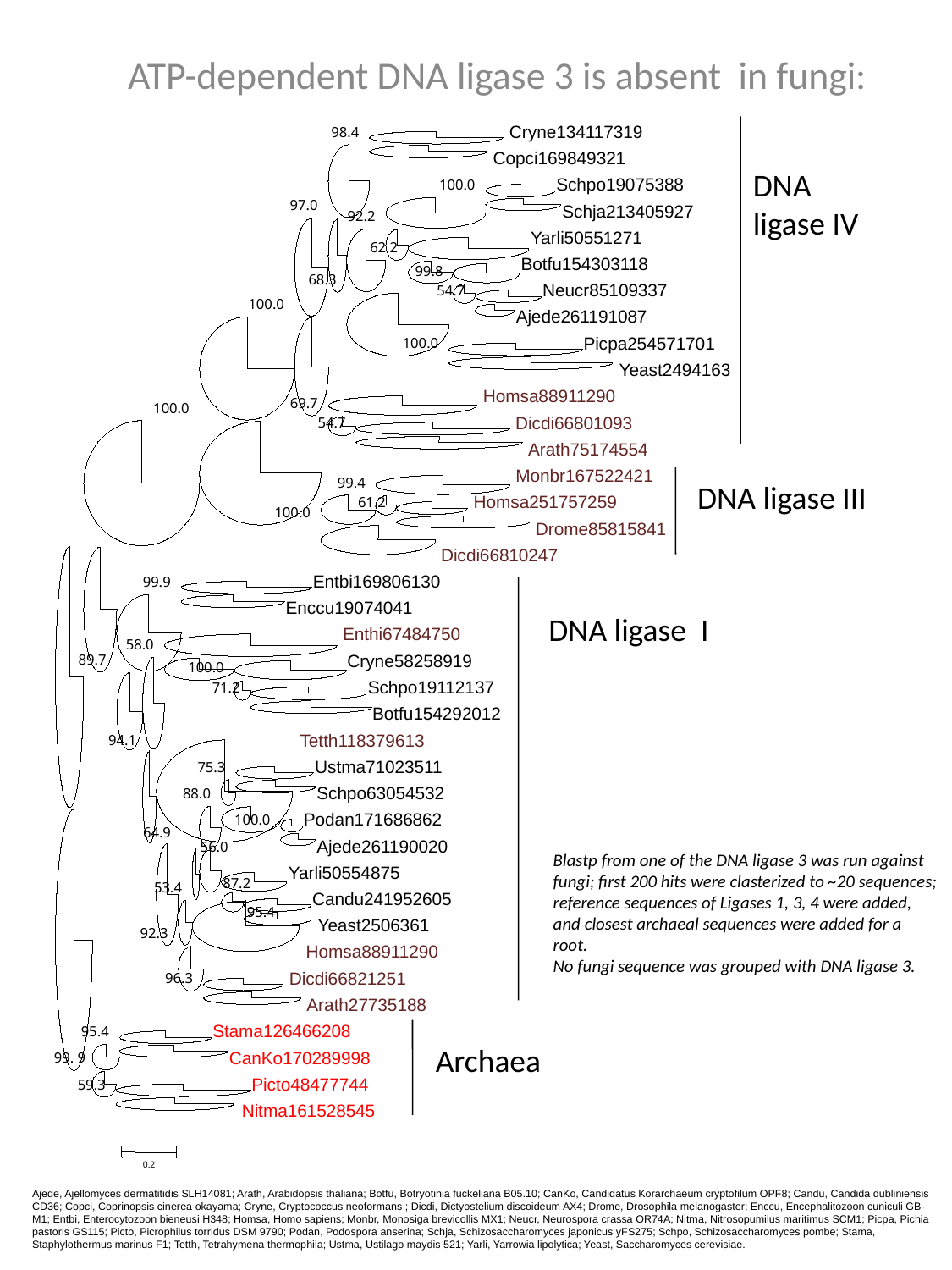

# ATP-dependent DNA ligase 3 is absent in fungi:
 Cryne134117319
98.4
 Copci169849321
DNA
ligase IV
Schpo19075388
100.0
97.0
Schja213405927
92.2
Yarli50551271
62.2
Botfu154303118
99.8
68.3
Neucr85109337
54.7
100.0
Ajede261191087
Picpa254571701
100.0
 Yeast2494163
 Homsa88911290
69.7
100.0
 Dicdi66801093
54.7
 Arath75174554
 Monbr167522421
DNA ligase III
99.4
 Homsa251757259
61.2
100.0
 Drome85815841
 Dicdi66810247
Entbi169806130
99.9
Enccu19074041
 DNA ligase I
 Enthi67484750
58.0
Cryne58258919
89.7
100.0
Schpo19112137
71.2
Botfu154292012
 Tetth118379613
94.1
Ustma71023511
75.3
Schpo63054532
88.0
Podan171686862
100.0
64.9
Ajede261190020
56.0
Blastp from one of the DNA ligase 3 was run against fungi; first 200 hits were clasterized to ~20 sequences; reference sequences of Ligases 1, 3, 4 were added, and closest archaeal sequences were added for a root.
No fungi sequence was grouped with DNA ligase 3.
Yarli50554875
87.2
53.4
Candu241952605
95.4
 Yeast2506361
92.3
 Homsa88911290
 Dicdi66821251
96.3
 Arath27735188
Stama126466208
95.4
Archaea
CanKo170289998
99. 9
Picto48477744
59.3
 Nitma161528545
0.2
Ajede, Ajellomyces dermatitidis SLH14081; Arath, Arabidopsis thaliana; Botfu, Botryotinia fuckeliana B05.10; CanKo, Candidatus Korarchaeum cryptofilum OPF8; Candu, Candida dubliniensis CD36; Copci, Coprinopsis cinerea okayama; Cryne, Cryptococcus neoformans ; Dicdi, Dictyostelium discoideum AX4; Drome, Drosophila melanogaster; Enccu, Encephalitozoon cuniculi GB-M1; Entbi, Enterocytozoon bieneusi H348; Homsa, Homo sapiens; Monbr, Monosiga brevicollis MX1; Neucr, Neurospora crassa OR74A; Nitma, Nitrosopumilus maritimus SCM1; Picpa, Pichia pastoris GS115; Picto, Picrophilus torridus DSM 9790; Podan, Podospora anserina; Schja, Schizosaccharomyces japonicus yFS275; Schpo, Schizosaccharomyces pombe; Stama, Staphylothermus marinus F1; Tetth, Tetrahymena thermophila; Ustma, Ustilago maydis 521; Yarli, Yarrowia lipolytica; Yeast, Saccharomyces cerevisiae.
