# Supplementary material for: Evolution of DNA ligases of Nucleo-Cytoplasmic Large DNA viruses of eukaryotes: a case of hidden complexity
Source: Biol Direct. 2009 Dec 18;4:51. doi: 10.1186/1745-6150-4-51 (PMC2806865; doi:10.1186/1745-6150-4-51)
Supplement: Additional file 4 — A maximum-likelihood phylogenetic tree of ATP-dependent DNA ligases including multiple vertebrate species. [file 1745-6150-4-51-S4.PPT]

## Slide 1
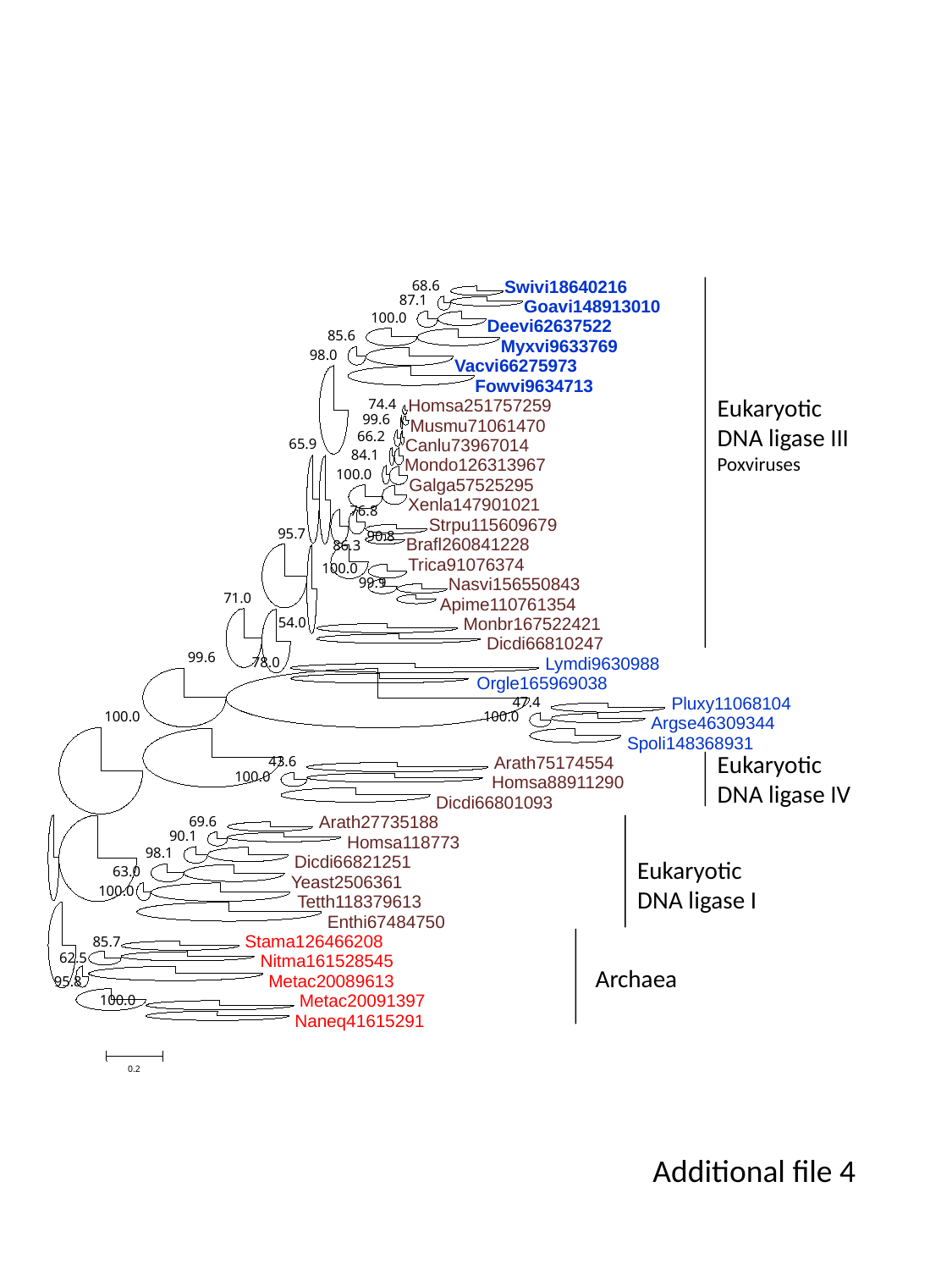

Swivi18640216
68.6
Eukaryotic DNA ligase III
Poxviruses
87.1
Goavi148913010
100.0
Deevi62637522
85.6
Myxvi9633769
98.0
Vacvi66275973
Fowvi9634713
Homsa251757259
74.4
99.6
Musmu71061470
66.2
Canlu73967014
65.9
84.1
Mondo126313967
100.0
Galga57525295
Xenla147901021
76.8
Strpu115609679
95.7
90.8
Brafl260841228
86.3
Trica91076374
100.0
Nasvi156550843
99.9
71.0
Apime110761354
 Monbr167522421
54.0
 Dicdi66810247
99.6
 Lymdi9630988
78.0
 Orgle165969038
 Pluxy11068104
47.4
100.0
100.0
 Argse46309344
 Spoli148368931
Eukaryotic DNA ligase IV
 Arath75174554
43.6
100.0
 Homsa88911290
 Dicdi66801093
 Arath27735188
69.6
Eukaryotic DNA ligase I
90.1
 Homsa118773
98.1
 Dicdi66821251
63.0
 Yeast2506361
100.0
 Tetth118379613
 Enthi67484750
Archaea
 Stama126466208
85.7
62.5
 Nitma161528545
 Metac20089613
95.8
 Metac20091397
100.0
 Naneq41615291
0.2
Additional file 4
#
